# Estimation of plasma apolipoprotein B concentration using routinely measured lipid biochemical tests in apparently healthy Asian adults

**DOI:** 10.1186/1475-2840-11-55

**Published:** 2012-05-18

**Authors:** Dong-Sik Cho, Sookyoung Woo, Seonwoo Kim, Christopher D Byrne, Joon-Hyuk Kong, Ki-Chul Sung

**Affiliations:** 1 Division of Cardiology, Department of Medicine, Kangbuk Samsung Hospital, Sungkyunkwan University School of Medicine, #108, Pyung Dong, Seoul, Jongro-Ku, 110-746, Republic of Korea; 2 Biostatistics Team, Samsung Biomedical Research Institute, Seoul, Republic of Korea; 3 Nutrition and Metabolism Unit, Faculty of Medicine, University of Southampton, IDS Building, Southampton General Hospital, Southampton, MP, 887, UK; 4 Department of Thoracic and Cardiovascular Surgery, Kangbuk Samsung Hospital, Sungkyunkwan University College of Medicine, Seoul, Korea

**Keywords:** Apolipoprotein, Formula, Hypercholesterolemia, Lipid, Cholesterol

## Abstract

**Background:**

Increased low-density lipoprotein cholesterol (LDL) concentration is associated with increased risk of coronary heart disease (CHD) but a substantial risk of cardiovascular disease often remains after LDL concentrations have been treated to target. Apolipoprotein B (apo B) is the major apolipoprotein contained within atherogenic lipoproteins such as LDL, and apo B is a more reliable indicator of cardiovascular risk than LDL concentration.

**Aim and methods:**

Our aim was to develop a formula for calculating apo B using lipid biochemistry measurements that are commonly available in clinical practice. We examined the clinical and laboratory data from 73,047 Koreans who underwent a medical health check that included apolipoprotein B concentration. The study sample was randomly divided into a training set for prediction model building and a validation set of equal size. Multivariable linear regression analysis was used to develop a prediction model equation for estimating apo B and to validate the developed model.

**Results:**

The best results for estimating apo B were derived from an equation utilising LDL and triglyceride (TG) concentrations [ApoB = −33.12 + 0.675*LDL + 11.95*ln(tg)]. This equation predicted the apo B result with a concordance correlation coefficient (CCC and 95%CIs) = 0.936 (0.935,0.937)).

**Conclusion:**

Our equation for predicting apo B concentrations from routine analytical lipid biochemistry provides a simple method for obtaining precise information about an important cardiovascular risk marker.

## Background

The association between increased concentrations of low-density lipoprotein cholesterol (LDL) and increased rates of premature coronary heart disease has been clearly demonstrated [1-8]. Current recommendations for the management of dyslipidemia are largely based on treatments to decrease LDL concentration [5,9-14]. However, a significant residual risk of cardiovascular disease (CVD) often remains after low-density lipoprotein cholesterol levels have been treated to target [15-20].

Apolipoprotein B100 (apoB) is the structural protein for atherogenic lipoproteins and facilitates the transporting of lipid from the liver to peripheral tissues [15,21-23]. A single apo B100 molecule is present in all major atherogenic particles derived from the liver (very low density lipoprotein (VLDL), intermediate density lipoprotein (IDL) and LDL). Consequently, measurement of apoB100 provides direct information as to the number of circulating atherogenic particles [23]. Apo B100 concentration is a better measure of LDL particle number concentration and is a more reliable indicator of risk than LDL concentration [22,24,25]. Thus, addition of apo B100 concentration to the routine lipid profile could improve identification of patients at risk of CVD and could improve management of those patients who are receiving lipid lowering therapy [24-31]. Apo B100 measurement also improves CHD risk prediction in people with diabetes or metabolic syndrome [24,32] and Apo B100 may provide a better assessment of on-treatment residual risk (than LDL) providing support for the notion that addition of apo B100 measurement to the routine lipid panel would improve patient management [26,31].

Apo B100 can be measured by commercial immunoassay [33] but assays are time-consuming and costly [34]. Although an algorithm for estimating apo B100 has previously been developed by Hermans et al. [23], this algorithm was developed in 45 people with diabetes from a Western population. Thus, the aim of our study was to develop an algorithm for estimating the apo B100 concentration from easily measured parameters; e.g. age, body mass index (BMI), low desntiy lipoprotein cholesterol (LDLc), high density lipoprotein cholesterol (HDLc), triglyceride (TG) and total cholesterol (TC) concentrations.

## Methods

A total of 73,047 apparently healthy subjects were recruited for the study. The mean age was 41.73 ± 8.4 years, [n = 44,118 men (41.9 ± 8.1-years) and n = 28,929 women (41.4 ± 8.7-years)]. Subjects participated in a routine health check-up program that was held at the Health Promotion Center of Kangbuk Samsung Hospital, Sungkyunkwan University School of Medicine, Seoul, Korea in 2008. The medical health checkup program was developed to improve the health of employees. Most subjects were employees, or family members, from various industrial companies across the country. The cost of medical examinations was predominantly paid for by the employers, and most subjects underwent a health check annually or biannually. The study protocol conformed to the ethical guidelines of the 1975 Declaration of Helsinki as reflected by a priori approval from our institution’s Human Research Committee.

The health check consisted of a full medical history and comprehensive blood test evaluation. Participants’ height and weight were measured barefoot and in light clothing. BMI was calculated as weight in kilograms divided by height in meters squared. Laboratory examinations were obtained after an overnight fast. An enzymatic calorimetric test was used to measure TC and TG concentrations. The selective inhibition method was used to measure HDLc, and a homogeneous enzymatic calorimetric method was used to measure the concentration of LDLc (Advia 1650 Autoanalyzer, Bayer Diagnostics, Leverkusen, Germany). Apo B100 and apoA1 concentrations were determined by rate nephelometry (IMMAGE system; Beckman Coulter).

Descriptive statistics for continuous variables are presented as means, standard deviations (SDs), medians and inter-quartile ranges (Q1, Q3). Categorical variable are presented as proportions (percentages).

The study sample was randomly divided into a training set for prediction model building and a validation set of equal size. Multivariable linear regression analysis was used to develop a prediction model equation and to validate the developed model. Natural log transformation was used to normalize the distribution of HDLc, TG, age and BMI. Analysis of residuals was used to check assumptions for multivariable linear regression modeling. The accuracy of the prediction model equation was evaluated using concordance correlation coefficient (CCC) analysis (Lin (1989)) that allowed comparison between prediction modeling results and the direct biochemical measurement of apo B100. In all tests, *p*-values < 0.05 were considered significant. Statistical analyses were performed using SAS 9.1.3 (SAS Institute Inc, Cary, NC) and R 2.13.2 (Vienna, Austria).

We conducted subgroup analyses by sex, glucose (7.0 mmol/l or 126 mg/dl), BMI (25kg/m^2^) and apoB quartile, in order to examine whether the derived equation was appropriate for specific subpopulation.

## Results

The characteristics of subjects in the model building subsample and the validation subsample were similar with no significant differences between groups (Table 1). Subjects with high glucose (>7.0 mmol/l, or >126 mg/dl) were only 932(2.6% of total subjects).Variables entered into the prediction equation were LDLc, HDLc, TG, age and BMI. LDLc, TC, ln(TG), ln(BMI) and ln(age) were each associated with apo B100, whereas apo B was not associated with HDLc.

**Table 1:** Characteristics of the whole cohort, and the model building and validation subsets

	**Total ** **(n = 73047)**	**Model building ** **(n = 36523)**	**Validation set ** **(n = 36524)**
Age (years)	41.73 ± 8.38	41.7 ± 8.4	41.7 ± 8.4
Gender (M/F)	44118/28929	22034/14489	22084/14440
Total cholesterol (mg/dL)	195.17 ± 33.79	195.12 ± 33.89	195.22 ± 33.70
TG (mg/dL)	127.55 ± 86.80	127.40 ± 86.25 104(73,156)	127.70 ± 87.34 105 (73,156)
HDLc (mg/dL)	55.20 ± 12.73	55.19 ± 12.73	55.20 ± 12.74
LDLc (mg/dL)	110.63 ± 29.43	110.62 ± 29.48	110.64 ± 29.37
ApoB100 (mg/dL)	97.52 ± 23.97	97.53 ± 24.04	97.50 ± 23.0
BMI (kg/m^2^)	23.63 ± 3.12	23.64 ± 3.12	23.63 ± 3.11

Data are expressed as mean ± SD or median [interquartile range], unless indicated otherwise. TG: triglyceride, HDLc: High density lipoprotein cholesterol, LDLc: low density lipoprotein cholesterol, Apo B100: apolipoprotein B100, BMI: body mass index.

We developed equations for predicting apo B100 from the results of multivariable regression modeling and compared apo B100 concentrations obtained by direct measurement with apo B100 estimates from various prediction model formulae (Table 2). An equation including LDLc, ln(TG), ln(age) and ln(BMI), produced the highest R^2^ results and the highest CCC. However, an equation that included LDLc, ln(TG) and ln(Age) produced a higher *F* –statistic than one that also included ln(BMI). However, since BMI added little to an equation that included just LDLc, and TG, we tested the equation ‘ApoB100 = −33.12 + 0.675*LDL + 11.95*ln(tg)’ in the validation data-set. In this data-set the CCC was 0.936(95% CI(0.935-0.937)).

**Table 2:** Models for predicting apoB100 concentration

	**Prediction equation**	**P-value**	**R-square**	**CCC[95% CI]**	
1. LDLc	ApoB100 = 16.177816 + 0.735235*LDLc	<.0001	81.1%	0.90	[0.890-0.898]
2. LDLc, age	ApoB100 = −24.77 + 0.72*LDLc + 11.43*ln(age)	<.0001	81.9%	0.90	[0.899-0.903]
3. LDLc, BMI	ApoB100 = −49.13 + 0.70*LDLc + 21.81*ln(BMI)	<.0001	82.4%	0.90	[0.902-0.905]
4. LDLc, BMI, age	ApoB100 = −78.91 + 0.696*LDLc + 9.81*ln(나이) + 19.98*ln(BMI)	<.0001	83.0%	0.91	[0.905-0.909]
5. LDLc, TG	ApoB100 = −33.12 + 0.675*LDLc + 11.95*ln(tg)	<.0001	88.0%	0.94	[0.935-0.937]
6. LDLc, TG, age	ApoB100 = −59.40 + 0.67*LDLc + 11.63*ln(tg) + 7.68*ln(age)	<.0001	88.3%	0.94	[0.937-0.939]
7. LDLc, TG, BMI	ApoB100 = −35.99 + 0.67*LDLc + 11.84*ln(tg) + 1.11*ln(BMI)	<.0001	88.0%	0.94	[0.935-0.937]
8. LDLc, TG, age, BMI	ApoB100 = −59.53 + 0.67*LDLc + 11.62*ln(tg) + 7.68*ln(age) + 0.05*ln(BMI)	<.0001	88.3%	0.94	[0.937-0.940]

→Multiple linear regression analyses were used to develop a prediction equation.

→CCC indicates concordance correlation coefficient.

→CI indicates confidence interval.

**Figure 1:**
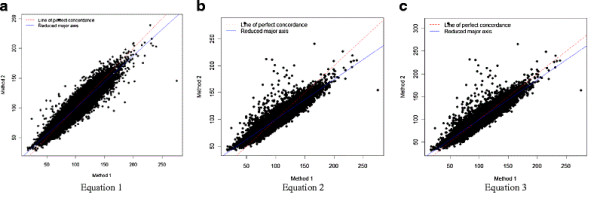
Scatter plot of estimated results by each equation and observed results.

We also estimated apo B100 using published prediction equations [23,33] and compared predicted values from these equations with concentrations obtained from direct biochemical measurements. Figures 1a, b and c show the scatter plots for the relationships between predicted and observed apo B measurements for the developed equation, and for the two published equations. We compared CCC values for our equation ApoB100 = −33.12 + 0.675*LDL + 11.95*ln(tg) with the two published equations (Table 3). We also compared the three equations stratified by sex, glucose (7.0mmol/l or 126 mg/dl) and BMI (≥25kg/m^2^) thresholds, and apo B100 quartiles (Table 4).

**Table 3:** CCC values for the developed equation* and published equations

	**CCC**	**[95% CI]**
Equation1: ApoB100 = −33.12 + 0.675*LDLc + 11.95*ln(tg)	0.94	[0.93 0.94]
Equation 2: ApoB100 = 20.67944 + 0.551614*non HDLc, where non HDLc = total C-HDLc	0.912	[0.91 0.91]
Equation 3: ApoB100 = 6.3 + 0.65*non HDLc, where non HDLc = total C-HDLc	0.93	[0.93 0.94]

Equation2: ApoB100 = 20.67944 + 0.551614*non HDLc, where non HDLc = total C-HDLc.

Equation 2 from Krishnaji Kulkarni (29).

Equation3: ApoB = 6.3 + 0.65*non HDLc, where non HDLc = total C-HDLc.

Equation 3 from Hermans et al(20).

**Table 4:** Comparative CCC results for the developed equation and two published equations, stratified by sex, glucose and BMI thresholds and apo B quartile

	**Male**			**Female**				**glucose >126mg/dl**				**Glucose <126mg/dl**			**BMI>25kg/m**^**2**^			**BMI<25kg/m**^**2**^		
	**CCC**	**[95% CI]**		**CCC**		**[95% CI]**		**CCC**	**[95% CI]**			**CCC**	**[95% CI]**		**CCC**	**[95% CI]**		**CCC**	**[95% CI]**	
Equation1	0.92	[0.92	0.93]	0.94		[0.93	0.94]	0.91	[0.90		0.92]	0.94	[0.93	0.94]	0.92	[0.91	0.92]	0.93	[0.93	0.94]
Equation2	0.90	[0.90	0.90]	0.91		[0.91	0.91]	0.84	[0.82		0.85]	0.91	[0.91	0.92]	0.89	[0.88	0.89]	0.91	[0.91	0.91]
Equation3	0.92	[0.92	0.92]	0.94		[0.94	0.94]	0.84	[0.82		0.86]	0.94	[0.94	0.94]	0.91	[0.91	0.91]	0.94	[0.94	0.94]
**Apolipoprotein B Quartile**																				
			**Q1(<80.24)**					**Q2(80.24< Q2< 90.95)**					**Q3(90.95< Q3<112.86)**				**Q4(>112.86)**			
			**CCC**		**[95% CI]**			**CCC**		**[95% CI]**			**CCC**		**[95% CI]**		**CCC**		**[95% CI]**	
Equation1			0.71		0.70		0.72	0.25		0.23		0.26	0.56		0.53	0.56	0.75		0.74	0.76
Equation2			0.51		0.50		0.51	0.22		0.20		0.23	0.56		0.55	0.57	0.72		0.71	0.72
Equation3			0.66		0.65		0.67	0.23		0.21		0.24	0.53		0.56	0.54	0.80		0.79	0.81

N = 36,524 participants who were randomly divided into a validation set.

The CCC for obese individuals with a BMI > 28kg/m^2^ was 0.914(95% CI(0.908-0.920)). We also tested the formula also in subjects with an atherogenic lipid profile, namely in those with a TG > 150 mg/dl + HDLc < 40/<50 mg/dl. In individuals with TG > 150 & HDLc < 50 (female), the CCC was 0.923(95% CI(0.917-0.929)). In individuals with TG > 150 & HDcL < 40 (male), the CCC was 0.932(95% CI(0.924-0.939)). We also calculated the performance of the proposed equation using estimated LDLc from Friedewald’s equation. The CCCs are 0.920(95% CI(0.919-0.922)) in the training data-set and 0.922(95% CI(0.920-0.924)) in the validation data-set.

## Discussion

Measurement of apo B100 concentrations helps cardiovascular risk prediction but unfortunately to date apo B100 assays are often not available because they are considered expensive and time consuming. Consequently, apo B100 measurements are not readily available to clinicians. From a very large cohort of subjects that are representative of a general Asian population, we have developed a very simple algorithm for estimating apo B100 concentration that utilizes only LDLc and fasting triglyceride concentrations. Although more complex equations fitted the data slightly better for predicting apo B100; the very simple algorithm did not compromise precision, compared with the more complex equations. We have also shown that the simple algorithm is valid in important sub groups of people that included obese subjects, subjects with increased plasma glucose concentrations and subjects with an atherogenic lipid profile. The derived formula is also appropriate if LDLc is estimated from Friedewald’s equation.

The CCCs for apoB100 in Q2 and Q3 were lower than for the other two quartiles. To evaluate the reason for the lower CCC results in Q2 and Q3, we compared the characteristics of the relationship between apoB100 and LDLc in each quartile. In the total data set, the relationship between apoB100 and LDLc showed a clear positive linear relationship, whereas in Q2 and Q3, the relationship was not linear In Q2 and Q3 the data points were concentrated around the middle of the distribution, rather than being spread evenly along the regression line. Consequently, the scatter of data in these middle two quartiles did not fit the regression line as well as in Q1 and Q4. Thus, this finding may limit the usefulness of our formula in these middle two quartiles. However, our equation was developed for the whole population and not just for the subjects in the 2^nd^ and 3^rd^ quartile. We reason that it is more important to fit the formula to the whole population and not to a specific subgroup within that population.

## Conclusion

In conclusion, we have developed an algorithm in a large Asian population to derive an estimate of apoB100 concetnrations that utilizes only measurement of LDLc and triglyceride concentrations from a fasting plasma sample. We are unable to comment as to whether the algorithm is valid in a Western population.

## Competing interests

All authors have no relevant conflicts of interests.

## Authors’ contributions

K-CS; study concept and design, acquisition of data; analysis and interpretation of data. D-SC; critical revision of the manuscript for important intellectual content. J-HK; critical revision of the manuscript for important intellectual content. SW; acquisition of data; analysis and interpretation of data. SK; acquisition of data; analysis and interpretation of data. CDB; critical revision of the manuscript for important intellectual content. All authors read and approved the final manuscript.
